# A Productive Proposed Search Syntax for Health Disaster Preparedness Research

**DOI:** 10.29252/beat-070201.

**Published:** 2019-04

**Authors:** Behnaz Rastegarfar, Ali Ardalan, Saharnaz Nejat, Abbasali Keshtkar, Mohammad Javad Moradian

**Affiliations:** 1 *Department of Disaster Public Health, School of Public Health, Tehran University of Medical Sciences, Tehran, Iran*; 2 *Department of Epidemiology and Biostatistics, School of Public Health, Tehran University of Medical Sciences, Tehran, Iran*; 3 *Department of Health Sciences Education Development, School of Public Health, Tehran University of Medical Sciences, Tehran, Iran*; 4 *Trauma Research Center, Shahid Rajaee (Emtiaz) Trauma Hospital, Shiraz University of Medical Sciences, Shiraz, Iran*

**Keywords:** Disaster, Health, Preparedness

## Abstract

**Objective:**

To find a proper search strategy to do a systematic review related to preparedness for disasters.

**Methods:**

MeSH and Emtree terms were searched to detect synonyms for two main search terms “disaster” and “preparedness”. Expert opinion on the synonyms was examined applying a Google form. The adopted syntax was searched in PubMed and results were sifted. Hand searching in two top key journals was done and sensitivity was calculated.

**Results:**

Out of 1120 articles, 122 were included. In PDM journal, 10 articles were included by hand searching, out of which 5 were not spotted in PubMed search with the proposed syntax. In DMPHP journal, 13 publications were included, with 5 not found in PubMed search. Because of human error in hand searching 2 articles were added.

**Conclusion:**

The proposed syntax in this study achieves a sensitivity of search of 0.6 in PubMed which could be quite applicable for researchers. Moreover, in case only MeSH or Emtree terms were applied in search strategy or where hand searching was not performed, there were a number of articles missed.

## Introduction

It is estimated that approximately half of the population of the world experienced a disaster between 2005 and 2015 with an unfortunate increase in the casualty and destruction intensity [1] . In view of these misfortunes, the necessity of enhancing preparedness for disasters to conduct the rescue operations most efficiently has been underscored, giving rise to further concentration on public health research on preparedness for disasters [2-5]. 

Literature review on the multifaceted and comparatively new field of health emergency management offers terms such as “response”, “preparedness”, “disaster”, “risk reduction”, and so on, none of which is as explicit or definite as desired [6,7]. The term “disaster” in itself has competing definitions: “emergency”, “incident”, “accident”, and “catastrophe” are the words that are interchangeably used in publications based on the authors’ ideas [8-10]. Some authors have used the word “readiness” and “preparedness” interchangeably [2, 11-21]. 

Zhong [22] states that preparedness is one of the key domains of resilience, Wachinger [2] and Norrisdiscuss [23] studied the relationship between preparedness and risk perception, Tosh [24] uses the term “*effective* preparedness” to reflect the necessity of the continuity of operations in health care and Bayntun [9] expresses that preparedness in emergency management includes functions that improve response capacity. He also defines preparedness as “the capability of the public health and health-care systems, communities, and individuals to prevent, protect against, quickly respond to, and recover from health emergencies, particularly those whose scale, timing, or unpredictability threatens to overwhelm routine capabilities”, which is close to the UNISDR’s definition that is used worldwide [25]. To simplify, no matter what phrase is used to define public health preparedness, it encompasses reducing vulnerability, increasing capacity, and thus being ready to respond and speed recovery [26]. This is while the results obtained from the MeSH search include the following terms: “Civil Defense”, “Defense, Civil”, “Defenses, Civil”, “Emergency Preparedness”, and “Preparedness, Emergency”.

One of the problems in literature review on disaster preparedness is the inconsistency of the applied labels. In this regard, Birnbaum *et al*., [7] have considered this inconsistency a result of the novelty of the field. In their classification of the relevant articles, they categorized the articles, in terms of their focus, into two major categories of epidemiological and interventional, with the latter including three subcategories of relief, recovery and risk reduction responses. Among essential requirements of any systematic literature review is having access to directions in literature search and particularly electronic search [27]. This is while in their research in 2017, Lefebvre *et al*., [28] stated that researchers tend not to mention their search methodology completely and precisely in their articles. They also maintained that could not spot any scientific article in which the researchers had explained their approach in selecting search filters. The aim of this study is to create a proper search strategy for a systematic review related to preparedness for disasters. This study is the first phase of a mixed method PhD thesis research in need of a tool capable of measuring health system preparedness for disasters. The results would also be applicable for disaster management researchers as well as databases’ administrator team. 

## Materials and Methods

In order to identify the best syntax, to be inclusive, two main search terms “disaster” and “preparedness” were created according to the objective of the study with an “all hazard” approach. MeSH and Emtree terms were searched to detect synonyms. Search terms were finalized according to the expert opinion.  Expert opinion on the synonyms for the terms “disaster” and “preparedness” was examined applying a Google form. The link of the Google form was sent to 10 experts who were associates of scientific committees of at least two international congresses on health in emergencies and disasters to come into more terminology which could be extracted to serve as a more efficient foundation for the search.

  The form included 2 questions: “1. which of the following words do you agree with for “preparedness” synonym? (Please insert your recommendation in the blank space.)”, and “2. which of the following words do you agree with for “disaster” synonym? (Please insert your recommendation in the blank space.)” Options in both questions were extracted according to a preliminary literature assessment by the research team and their priori knowledge. To be inclusive, even if proposed by only one of the experts, the recommended term was included in the syntax.

The basic syntax was adopted in PubMed until the proper NNR (number need to read) was achieved (12.5). Applying two PubMed tags, [sb] and [pt], “systematic [sb]” and “review [pt]” were added to the basic syntax separately to restrict the huge bulk of the original articles. Because these two tags are very sensitive in PubMed, the search result would be secondary articles with the proposed syntax.

Each final syntax was searched in PubMed in July 2017 and was updated until the 16th of Aug 2017 . All records were imported to EndNote software (Reuters T. EndNote X7. Thomson Reuters: Philadelphia, PA, USA. 2013). after removing duplicates; titles, abstracts, and full texts (if needed) were reviewed by the first author (BR). In case of doubt, MJM and AA were the consultants. For the second step, key journals were examined as a validation set. To identify top key journals, the adopted syntax was searched in Scopus. Hand searching, with the same inclusion and exclusion criteria, from 1990 to Aug 2017, was done in two top key journals. Google translator was applied for non-English articles.

The inclusion and exclusion criteria were as follows, using the high sensitive approach. Papers were expected to meet all of these criteria.

- Inclusion:

- Related to disasters and emergencies
- Peer-reviewed secondary studies [29]
- Directly addressing preparedness for disasters in the health system.
- For the purposes of this review, “disaster” and “preparedness” are defined as follows:

- Preparedness: the aggregate of all measures and policies adopted by the health system before an event occurs that promotes mitigation of the damage caused by an event and minimizes the dysfunction that could result from the damage [30].
- Disaster: serious condition beyond the normal capacity of the local community to cope, thereby, justifying external assistance [31].

- Exclusion

- Published before 1990
- Full text not available
- Addressing preparedness not in the human health system
- Articles doing general literature review or overview without mentioning explicit search strategy and methodology
- Secondary studies reviewing reports, guidelines, plans or apps
- Addressing response or recovery in disasters even if the results could be useful for preparedness

 “Relative Recall” was calculated to discuss the appropriateness of the recommended strategy and refer to the sensitivity index. We defied relative recall as: 

Number of included secondary articles retrieved by the search strategy in PubMed 

Relative Recall 

Number of included secondary articles spotted by hand searching in two top key journals 

## Results

*Syntax development*

 For “disaster” in MeSH search, the results were:

- Natural Disasters
- Disaster, Natural
- Disasters, Natural
- Natural Disaster

While in Emtree it was “catastrophe”; also in keeping with the expert idea the terms “incident”, “crisis”, “emergency”, and “accident” were added to the synonym list for “disaster”.

For “preparedness” synonyms, experts stated “readiness”, however, there was no synonym in Emtree. Results for synonyms are demonstrated in Table 1. Proposed syntax with the exact spelling and combinations found in MeSH, Emtree, and the experts’ suggestions was developed in PubMed as follow:(Disaster OR “Natural Disasters” OR (Disaster* AND Natural) OR “Natural Disaster” OR catastrophe OR incident OR crisis OR emergency OR accident) AND (“Civil Defenses” OR (Defense* AND Civil) OR “Emergency Preparedness” OR (Preparedness AND Emergency) OR readiness OR preparedness) AND 1990:2017[dp] 

**Table 1:** Results for synonyms of “disaster” and “preparedness” in MeSH, Emtree and expert opinion survey

**Source of key words**	**Disaster**	**Preparedness**
**MeSH**	Natural DisastersDisaster, NaturalDisasters, NaturalNatural Disaster	Civil DefensesDefense, CivilDefenses, CivilEmergency PreparednessPreparedness, Emergency
**Emtree**	Catastrophe	---
**Expert opinion**	IncidentCrisisEmergencyAccident	Readiness

*Secondary studies in PubMed*

 Out of 1120 articles, 122 (10.89%) were included. Results of the final syntax search in PubMed is shown in Table 2. Agreement between two reviewers for which articles met all scientific criteria was 81% (kappa statistic, CI 95: 0.79, 0.84).

**Table 2:** Result of syntaxes searched in PubMed (updated 16th Aug 2017)

**Syntax**	**Number of records**
systematic[sb] AND ((Disaster OR “Natural Disasters” OR (Disaster* AND Natural) OR “Natural Disaster” OR catastrophe OR incident OR crisis OR emergency OR accident) AND (“Civil Defenses” OR (Defense* AND Civil) OR “Emergency Preparedness” OR (Preparedness AND Emergency) OR readiness OR preparedness) AND 1990:2017[dp])	282
review[pt] AND ((Disaster OR “Natural Disasters” OR (Disaster* AND Natural) OR “Natural Disaster” OR catastrophe OR incident OR crisis OR emergency OR accident) AND (“Civil Defenses” OR (Defense* AND Civil) OR “Emergency Preparedness” OR (Preparedness AND Emergency) OR readiness OR preparedness) AND 1990:2017[dp])	838

*Key journal hand searching*

 The adopted syntax in Scopus resulted in 76065 articles. After the 29th round of adaptation, NNR=14.2 was reached with 2864 articles. In this round, the two top key journals were “Prehospital and Disaster Medicine” (PDM) and “Disaster Medicine and Public Health Preparedness” (DMPHP). Out of 28 volumes of PDM journal from 1990s, 10 articles were included, out of which 5 were not spotted in PubMed search with the proposed syntax. In DMPHP journal, Since July 2007, 11 volumes were available, from which 13 publications were included, with 5 (38.5%) not found in PubMed search.

In each journal, there was one article which was not detected in hand searching but included in PubMed search because of human error. Totally there were 25 articles included in hand searching, 15 of which were duplicated in comparison with the PubMed search. The title of articles which were found only by hand searching are noted in Table 3. The relative recall was 0.6 (CI 95: 0.40, 0.79) in this case.

**Table 3:** Title of articles found in key journals (PDM and DMPHP) by hand searching

	**PDM**	**DMPHP**
**1**	Characteristics of Medical Teams in Disaster.	Core Competencies in Disaster Management and Humanitarian Assistance: A Systematic Review.
**2**	Mass-Gathering Medical Care: A Review of the Literature	Hospital Referral Patterns: How Emergency Medical Care Is Accessed in a Disaster.
**3**	Disaster Preparedness among Health Professionals and Support Staff: What is Effective? An Integrative Literature Review.	Improving Long-Term Care Facility Disaster Preparedness and Response: A Literature Review.
**4**	Enhancing the Minimum Data Set for Mass-Gathering Research and Evaluation: An Integrative Literature Review.	Review of Hospital Preparedness Instruments for National Incident Management System Compliance.
**5**	Estimation of the Demand for Hospital Care After a Possible High-Magnitude Earthquake in the City of Lima, Peru. (“civil defense” & disaster in abstract)	Defining Roles for Pharmacy Personnel in Disaster Response and Emergency Preparedness.

## Discussion

 This study is the cornerstone of a systematic review to synthesize the existing literature on public health preparedness measurement for disasters that will guide future work on intervention development in this field. Countries and their crisis management departments constantly seek to enhance their capability of confronting disasters through disaster preparedness plans, this is while they are in short of even a uniformly-agreed-upon definition for the terms “disaster” and “preparedness”, or any confirmed response performance measures [4]. This is while multi-disciplinary characteristics of disasters raise various interpretations and proposes definitions that reflect each individual discipline’s interests [31]. The concepts of response and preparedness are intertwined; performing preparedness measures might result in an improved response, while the experience gained from the response might bring about superior preparedness. As a result, it is probable that in articles considered by the authors to be focused on preparedness, actually the response to a disaster, the gained experience, and the disaster characteristics have been presented, or the requirements of an appropriate response may have been investigated [32]. Nevertheless, as a result of the dispersal of applicable articles, the searching process can be highly demanding. Additionally, many users lack the required skill to search databases. The factor of the indexing limitations also adds to the search challenges [33]. The objective regarding this situation is to document a proper syntax to investigate the literature related to health disaster preparedness. 

Birnbaum *et al*., [7] have analyzed health disaster publications and like the present study have investigated two health disaster related journals, PDM and DMPHP, as two journals comprising the bulk of health disaster management articles. While they do not have any category belonging to preparedness, it seems that this research is included in the Epidemiological category. 

The synonyms for “disaster” proposed by the experts had been employed in most articles; in case of merely using MeSH terms, the proposed syntax was not inclusive enough (24 out of 122 included both labels “disaster” and “natural”). With regard to the synonyms for “preparedness” spotted in MeSH, out of 122 articles only 2 had used “defense” in their abstracts, which even did not intend to denote preparedness. The term “civil” had been employed in three abstracts. The terms “civil” and “defense” had been used together in 12 articles.

On the other hand, the methodological structure of the studies was not uniform, for example a secondary study might not be mentioned as a secondary in its title or abstract so one cannot rely only on the title or abstract. Even after the publication of PRISMA guideline in 2009, only 4 articles had applied the guideline. This might be the reason why in spite of Birnbaum *et al*., [7] statement that no systematic review was published in PDM journal from 2009-2014, it was spotted some in the PubMed search with the proposed syntax [34-36].

The analysis of the 10 articles found by hand searching shows that only one article has not employed any of the keywords used in the proposed syntax, however, it’s abstract contained two keywords: “civil defense” and “disaster” (Table 3). PubMed search sensitivity might be the reason why these articles are not being included in the syntax search. Surely, MEDLINE is one of the main resources in the development and maintenance of an evidence-based approach to disaster management that indexes approximately 75% of all peer-reviewed, event-specific literature [31]. 

Researchers focusing on a new systematic review or looking for updates on certain topics, who as an element of their inclusive criteria employ a method filter, would mainly benefit from the most sensitive search, although they may come up with loads of irrelevant articles and reviews. Searchers wish for searching instruments which are precise and responsive enough to turn in results which are as relevant as possible, while excluding as many irrelevant data as possible. To this purpose, searching methods are most applicable which focus on the highest levels of sensitivity and specificity, but a low level of difference, which, however, does not eliminate the later need for discarding irrelevant results [32]. While in this study the sensitivity of PubMed search with specified tags ([sb] and [pt]) has achieved 60%, Egan *et al*., [26] express that applying generic terms and consequently achieving highly sensitive searches would not result in inclusive and high quality results. A common problem of search results in non-clinical fields is a bulk of irrelevant results which follow from a shortage of technical terms, such as those used in the clinical- fields, as well as employing non-technical terms in search operations [27].

Hence, in case of health disaster management reviews, it would be difficult to have greater precision without compromising sensitivity. Adopting strategies for applying definite guidelines for the structure and writing of articles might obtain greater precision. Researchers can also obtain higher degrees of precision through applying “AND” and “AND NOT” operators. Applying these operators alongside relevant technical terminology can help to promote search results to a great extent [32]. 

To sum up, the proposed syntax in this study, “(Disaster OR “Natural Disasters” OR (Disaster* AND Natural) OR “Natural Disaster” OR catastrophe OR incident OR crisis OR emergency OR accident) AND (“Civil Defenses” OR (Defense* AND Civil) OR “Emergency Preparedness” OR (Preparedness AND Emergency) OR readiness OR preparedness) AND 1990:2017[dp]”, achieves a sensitivity of search of 0.6 in PubMed which could be quite applicable for researchers. Moreover, in case only MeSH or Emtree terms were applied in search strategy or where hand searching was not performed, there were a number of articles missed. Hence, measures to increase the sensitivity of the search results are as follows: developing definite meanings for health disaster management terminology, revising MeSH and Emtree terms, and adopting new strategies to emphasize on applying unified structures by the authors. Creating technical terms for the field of health disaster management would be of great value. Collectively, all these measures would make the result as effective as desired.

The limitations to this study are as follows: First, the approach that this article is following is an “all hazard” one, therefore there is no requirement for including specific hazards such as flood, fire or any other natural or manmade disasters. In case, other researchers need to focus on any specific hazard or alternative spelling/combination, they can simply do it by using “AND” and “OR” operators. Secondly, syntax search was performed only in PubMed, consequently, specific tags for systematic reviews and reviews are available only in PubMed. Obviously, using the original articles would result in a bulk of articles whose review would call for a budget of time disproportionate with the scope of this study. Therefore, the approved labels [sb] and [pt] were applied to spot the secondary related articles. If any researcher wishes to use the original articles, it will be possible through using our suggested syntax. Thirdly, hand searching was performed only in two top of the current, peer-reviewed health disaster journals. Fourthly, hand searching in DMPHP journal was available only from 2007; and fifthly, human error in hand searching mostly because of lacking a unified structure.

## Conflict of interest:

No conflict of interest. 
